# Supplementary material for: Learning data-efficient coarse-grained molecular dynamics from forces and noise
Source: Nat Commun. 2026 Mar 15;17:2493. doi: 10.1038/s41467-026-70818-0 (PMC12992897; doi:10.1038/s41467-026-70818-0)
Supplement: Supplementary file 1 — Supplementary Information [file 41467_2026_70818_MOESM1_ESM.pdf]

Supplementary Information for: Learning data efficient coarse-grained molecular dynamics from forces and noise

Aleksander E. P. Durumeric[1] and Yaoyi Chen[1]

*Department of Mathematics and Computer Science,
Freie Universität Berlin, Arnimallee 12, 14195 Berlin, Germany*

Aldo S. Pasos-Trejo[1]

*Department of Physics, Freie Universität Berlin,
Arnimallee 12, 14195 Berlin, Germany*

Frank Noé*

*Department of Mathematics and Computer Science,
Freie Universität Berlin, Arnimallee 12, 14195 Berlin, Germany*

*Department of Physics, Freie Universität Berlin,
Arnimallee 12, 14195 Berlin, Germany and*

AI for Science, Microsoft Research, Karl-Liebknecht Str. 32, 10178 Berlin, Germany

Cecilia Clementi*

*Department of Physics, Freie Universität Berlin,
Arnimallee 12, 14195 Berlin, Germany*

*Center for Theoretical Biological Physics,
Rice University, Houston, 77005, TX, USA and*

Department of Chemistry, Rice University, Houston, 77005, TX, USA.

CONTENTS

I. Theory 2
A. Noise and the mb-PMF 2
B. Ensemble averages via noising 4
C. Optimization of force aggregation for coarse-graining 5

II. Additional numerical results 5
A. Force maps that incorporate both forces and noise 5
B. Model training 6
1. Model selection 7
2. Convergence of model simulations 8
C. Two-dimensional FESs 10
D. Results for Chignolin 13
E. Rare-event sampling 13

References 18

I. THEORY

The equations in the main text and this supplementary information apply to a system of n atoms with coordinates $\mathbf{r} \in V \subset \mathbb{R}^{3n}$ in the canonical ensemble. The CG resolution is defined using the full rank matrix $\mathbf{M}$ which projects the atomistic coordinates $\mathbf{r}$ to their coarse-grained (CG) counterparts $\mathbf{Mr} = \mathbf{R} \in \mathbb{R}^{3N}$ comprising N "beads" with $N \ll n$. Both the atomistic and CG force-fields are described here in thermal units ($\beta = 1$), and the two systems are set to have the same temperature in practice. We only consider configurational consistency (and not momentum consistency) [1].

A. Noise and the mb-PMF

As stated in the main text, combining noise with atomistic force information can be described using a function referred to as the noised mb-PMF. This surface may be mathematically understood using tools central to bottom-up coarse-graining, which typically creates CG force-fields approximating the (unnoised) mb-PMF [2, 3]. The unnoised mb-PMF (U_{PMF}) is defined (up to a constant) as:

$$U_{\mathrm{PMF}}(\mathbf{R}) = -\log \int \delta[\mathbf{R} - \mathbf{Mr}] \exp\left[-u(\mathbf{r})\right] \mathrm{d}\mathbf{r} \tag{S1}$$

where integration is applied over the entire domain of the corresponding probability distribution. Eq. (S1) may be modified by replacing the Dirac δ with a kernel function κ. Unlike δ, κ is not defined solely as a distribution, but is instead a positive scalar-valued function with well-defined log gradients with respect to all arguments. Given its relationship to δ, intuitive κ are concentrated where $\mathbf{R}$ is close to $\mathbf{Mr}$ for a predefined $\mathbf{M}$; in the experiments of this work, κ is defined to be a multivariate Gaussian density over $\mathbf{R}$ centered at $\mathbf{Mr}$ with a covariance matrix proportional to the identity matrix. This substitution results in a "smoothed" or noised variant of the mb-PMF ($U^{\kappa}_{\mathrm{PMF}}$) defined as:

$$U^{\kappa}_{\mathrm{PMF}}(\mathbf{R}) = -\log \int \kappa[\mathbf{R}, \mathbf{r}] \exp\left[-u(\mathbf{r})\right] \mathrm{d}\mathbf{r}. \tag{S2}$$

Critically, as κ is strictly positive, the integration against κ present in Eq. (S2) may be itself understood as a traditional coarse-graining problem, where our CG configurational map is defined to be

$(\mathbf{r}, \mathbf{R}) \mapsto \mathbf{R}$ (isolation of $\mathbf{R}$):

$$\int \kappa[\mathbf{R}, \mathbf{r}] \exp\left[-u(\mathbf{r})\right] \mathrm{d}\mathbf{r} = \int \delta[\mathbf{R} - \mathbf{R}'] \exp\left[-u(\mathbf{r}) + \log \kappa(\mathbf{R}', \mathbf{r})\right] \mathrm{d}\mathbf{r}\mathrm{d}\mathbf{R}'. \tag{S3}$$

These equations interpret the effect of κ through an induced force-field term $\log \kappa(\mathbf{R}', \mathbf{r})$ with $\mathbf{R}'$ part of an extended configurational state. This view may in turn be combined with established literature to understand rigorous requirements on κ [4, 5]; we note that for all κ discussed in the main text, the corresponding $\log \kappa$ are harmonic potentials in the CG space, centered on $\mathbf{Mr}$. As Eq. (S2) illustrates, traditional non-noised coarse-graining expressions are regained via the limiting case of Gaussian κ with vanishing variance, although Eq. (S3) then becomes undefined.

As Eq. (S3) represents a typical coarse-graining operation, it may be analyzed through the lens of force-matching (multiscale coarse-graining) [1], giving rise to Eq. (3). However, it is important to remark that the gradient operation typically applied to u to define forces must instead be applied to $u - \log \kappa$ and taken with respect to both $\mathbf{r}$ and $\mathbf{R}$; this operation defines forces on the κ-induced particles $\mathbf{R}$ and modifies the forces on the "real" particles $\mathbf{r}$; in the main text, the corresponding gradient domain is made clear by context. The combination of the implied CG map and modified forces, when combined with existing framework relating transformed atomistic forces to their CG counterparts [1, 4, 6, 7], implies the various $\mathbf{T}$s utilized in the main text. In particular, mapping the forces to retain only the forces on the configurationally preserved particles results in $\mathbf{T}_{\text{noise}}$, as u is *not* a function of the preserved particles.

$$\begin{aligned} \mathbf{T}_{\text{noise}} f(\mathbf{R}, \mathbf{r}) &= \mathbf{T}_{\text{noise}} \left[-\nabla u(\mathbf{r}) + \nabla \log \kappa(\mathbf{R}, \mathbf{r})\right] \\ &= -\nabla_{\mathbf{R}} [u(\mathbf{r}) - \log \kappa(\mathbf{R}, \mathbf{r})] \\ &= \nabla \log \kappa(\mathbf{R}, \mathbf{r}) \end{aligned} \tag{S4}$$

The extended phase space viewpoint described in Eq. (S3) allows corrections related to constrained bonds to be incorporated in a straightforward way using existing work [6, 7]; all derived $\mathbf{T}$ used in this work respect these conditions (see also section I C below). We further note that analysis related to the thermodynamic state may be performed as long as choices are made with regard to the state-dependence of κ [2, 3]. Replacement of Dirac deltas with Gaussian functions has been historically used in the derivation of multiple methods typical to enhanced sampling such as umbrella sampling [8], adiabatic free energy dynamics [9, 10], and extended adaptive biasing force [11, 12], providing intriguing avenues for further analysis.

As where minimization of Eq. (1) results in U_{PMF}, minimization of Eq. (3) results in U^{κ}_{PMF}. These are not generally the same function; however, as shown in the main text, κ may be chosen such that they are very similar. In this view the expressions discussed in the main text introduce a *bias* into the integral form of the learning objective [13]; however, as numerically demonstrated, finite-sample training procedures derived from these biased objectives may better reproduce the metastable behavior in the unnoised mb-PMF than their unmodified counterparts. This property motivates selecting κ (e.g., through σ) in the same way as is done for typical hyperparameter selection: various κs are attempted, and an optimal one is selected based on a given validation metric.

We note that while the above perspective considers the unnoised mb-PMF to be the ultimate goal due to its theoretical properties [2, 3], the noised mb-PMF may also represent an important object in its own right if it corresponds to a smoother surface allowing for accelerated sampling. From this perspective, κ may be viewed not simply as a stabilization to improve learned approximations to mb-PMF, but rather as a fundamental redefinition of the relationship between the representation of the learned model and the fine-grained system (Eq. (S2)). This viewpoint suggests that κ should be selected so that the noised mb-PMF is both physically meaningful and corresponds to a viable learning objective given the available reference data and force-field parameterization; future optimization of the selection of κ will be pursued in later work.

Note that when applied to non-equilibrium data, κ and $\mathbf{T}$ together control a trade off between force-based and distributional information. Empirically, when $\mathbf{T}$ is defined by minimizing force fluctuations, more diffuse κs tend to have larger contributions to the training signal created by the application of $\mathbf{T}$; as a result, it is likely that more diffuse κs impart a larger level of distributional bias.

If $\kappa(\mathbf{R}, \mathbf{r}) := \phi[\mathbf{R} - \mathbf{M}\mathbf{r}]$ for a suitable function ϕ, algebraic operations typically directly performed on δ in Eq. (S1) (e.g., chain rule and integration by parts [1, 6]) may similarly be performed on κ in Eq. (S2). This connection may be used to create training forces corresponding to the noised ensemble that do not utilize the log gradients of κ, but rather only use contributions from u, demonstrating that the log gradient of Eq. (S2) may be defined through averages of atomistic forces. More broadly, as in the case of unnoised force-matching [7], many possible $\mathbf{T}$ operators are compatible with the integration in Eq. (S3). Certain maps, such as $\mathbf{T}_{\text{noise}}$, only utilize force information associated with a particular set of particles; others, such as those used when combining force and noise information, may be selected for favorable learning properties.

B. Ensemble averages via noising

In the case of traditional force-matching, the ensemble average in Eq. (1) is approximated using samples from atomistic MD. However, the expectation in Eq. (3) over the extended system defined in Eq. (S3) is not directly available from our reference simulations. In this work we approximate these extended ensemble averages by combining atomistic MD with ancestral sampling [14]. In general, ancestral sampling draws variates from a joint probability density function $f(x, y)$ by first sampling x from its marginal distribution $f_X := \int f(\cdot, y)\mathrm{d}y$ and then sampling y via its conditional probability $f(x, \cdot)/f_X(x)$. This may be applied to the extended ensemble by first noting that said ensemble is governed by probability density $p \propto \kappa \exp[-u]$. If $\int \kappa(\mathbf{R}, \mathbf{r})\mathrm{d}\mathbf{R}$ is independent of $\mathbf{r}$, the marginal density over $\mathbf{r}$ remains proportional to $\exp[-u]$ and we may interpret $\kappa(\mathbf{R}, \mathbf{r})$ to be proportional to the conditional probability of $\mathbf{R}$ given $\mathbf{r}$. In this case we may use the following procedure to generate samples from the extended ensemble:

1. Generate n samples from $\exp[-u]$, denoted $\mathbf{r}_i$
2. Given each sample $\mathbf{r}_i$, sample $\mathbf{R}_i \sim \kappa(\cdot, \mathbf{r}_i)$ to create $(\mathbf{r}_i, \mathbf{R}_i)$

When $\exp[-u]$ corresponds to the equilibrium density of the atomistic molecular dynamics simulation and $\kappa(\mathbf{R}, \mathbf{r}) \propto \exp[-(\mathbf{M}\mathbf{r} - \mathbf{R})^{\intercal}\Sigma^{-1}(\mathbf{M}\mathbf{r} - \mathbf{R})]$ for suitable covariance matrix Σ, this procedure describes the process of injecting Gaussian noise: We first generate atomistic configurations and subsequently combine them with Gaussian noise to create an extended ensemble containing both the noised and unnoised variables.

Correlation-based force-field training strategies (e.g., [15, 16]) directly utilize the distribution of configurations to determine a force-field. In the case of unnoised coarse-graining, mapping samples from an equilibrium atomistic simulation produces configurations drawn from the mb-PMF. When applied to equilibrium atomistic samples, the above κ-based sampling procedure similarly produces samples which, when mapped to isolate the κ-induced particles, correspond to the noised mb-PMF. As a result, correlation-based training techniques may be combined with noised samples in the same way as is done for non-noised coarse-graining [17]. This property allows the prior energy terms present in the utilized force-field architecture [18] to be calculated via histograms of noised samples at each noise level. As these prior terms are represented by fixed functional forms, high noise may create distributions that are difficult for the priors to approximate; this may be the cause of breakdown at higher noise levels. The strong relationship between noise-derived forces and the resulting empirical correlations may provide interesting extensions to previous unifications

of force and correlation information [15, 19, 20]. Our numerical results on mixing force and noise information demonstrate that when applied to non-equilibrated samples from atomistic simulation, low variance noise combined with atomistic forces information approximately maintains the ability of force-matching to learn the underlying mb-PMF from non-equilibrium samples. Partial insight into this phenomenon can be seen by log-differentiating Eq. (S2) in the same manner as in previous work [1, 6], showing that locality in the definition of κ in turn induces locality in the contribution of atomistic forces.

C. Optimization of force aggregation for coarse-graining

The force mapping $\mathbf{T}$ in Eq. (1) must satisfy two theoretical constraints [6, 7]:

- The mapped forces must not include components that act along rigid constraints (e.g., constrained bonds). Otherwise, the CG force field becomes physically incorrect.
- The force mapping $\mathbf{T}$ must be mathematically compatible with the mapping $\mathbf{M}$ of the atomistic, $\mathbf{r}$, to CG coordinates, $\mathbf{R} = \mathbf{M}\mathbf{r}$, that is: $\mathbf{T}\mathbf{M}^T = \mathbf{I}$.

In practice, the above constraints still allow for many possible consistent force mappings. In particular, all the force map coefficients corresponding to atoms that are not identified with or constrained to any CG bead can take arbitrary real values. This freedom in the choice of the force map can be exploited to maximally reduce the noise in the force matching residual in Eq. (1). It was shown [7] that this optimization greatly improves the accuracy and data efficiency of machine-learned coarse-grained molecular dynamics models.

In the case of a linear coordinate map $\mathbf{M}$ and particle-specific force contributions, this optimization of the force map $\mathbf{T}$ reduces to an independent smoothing optimization statement for each CG site I:

$$\min \|\eta_I \mathbf{C}\underline{\mathbf{F}}\|_2^2 \tag{S5}$$

where $\underline{\mathbf{F}} \in \mathbb{R}^{n\times 3n_t}$ contains reshaped atomistic forces present in a molecular trajectory with n_t frames, $\mathbf{C} \in \{0,1\}^{|\eta_I|\times n}$ is a sparse matrix representing the molecular constraints present in the atomistic system, and η_I is a real (row) vector describing the parameterization of $\mathbf{T}$ corresponding to single CG site I. The optimization of Eq. S5 can be solved in a straightforward manner using linearly-constrained quadratic programming.

II. ADDITIONAL NUMERICAL RESULTS

A. Force maps that incorporate both forces and noise

As mentioned in the main text, the force maps for combining both atomistic and noise forces are optimized with the `stagedjoptgauss_map` and `stagedjslicegauss_map` methods of the aggforce package [21]. Practically, this involves two steps. Firstly, a conventional CG force map is optimized to project the all-atom forces onto the CG resolution $\mathbf{M}\mathbf{r}$. We omit here the process and outcome of this step since it yields exactly the same result as for the maps used for force-matching-only MLCG models, which can be found in Ref. [7] (see also section I C above). Subsequently, a secondary CG map is defined to slice the $\mathbf{R}$ out of the pairs $(\mathbf{M}\mathbf{r}, \mathbf{R})$ sampled according to the definition of κ.

The force map $\mathbf{T} \in \mathbb{R}^{2N\times N}$, where N is the number of CG beads, is optimized from data (example for Trp-Cage in Fig. S1) and is a function of the noise level σ^2. The right half of the force map matrix is always an identity matrix due to the definition of a valid force map [7] (see section I C). The left half, corresponding to the contribution of atomistic forces, depends on the noise level. We observed

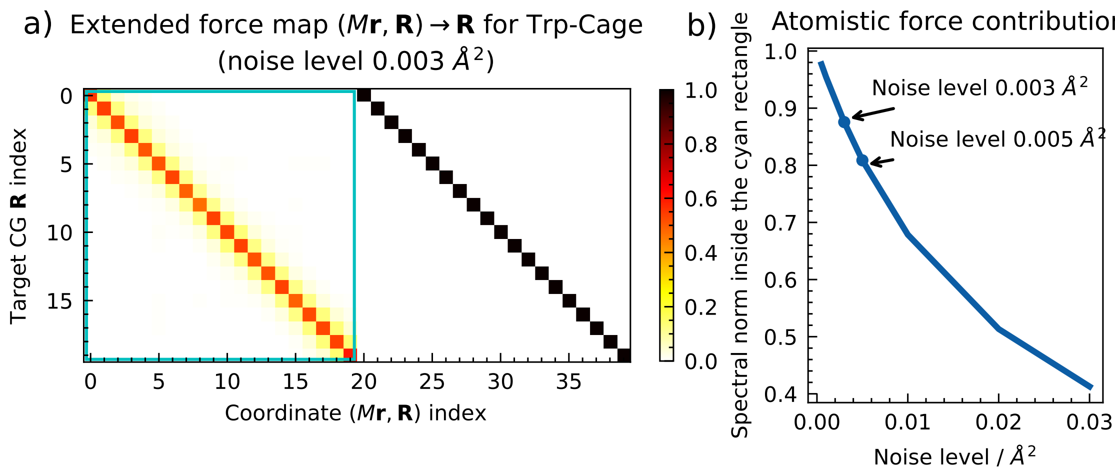

FIG. S1. Noise-force map $\mathbf{T}$ for Trp-Cage at different noise levels. a) Example of noise-force map at noise level $\sigma^2 = 0.003$ Å^2. The left half panel surrounded by a cyan rectangle corresponds to the contribution from the atomistic forces (via the first-stage force map), while the right part corresponds to the noise contribution. b) The magnitude of the atomistic force influence, measured by the spectral norm (i.e. the largest singular value) of the square submatrix in the cyan rectangle of panel a). The decreasing value as function of the noise level shows the trade-off between the atomistic force and the noise force contributions.

that, in this left half submatrix, only the near-diagonal elements are non-zero and that the largest entries correspond to the contribution from the "real" CG particles, to which the noised CG bead is tethered, making the atomistic portion of the force map matrix nearly diagonal. The magnitude of these coefficients, which measure the influence of the atomistic force in the total final force, depends on the noise level. We can quantify the magnitude of this influence by calculating the spectral norm of the left submatrix, which corresponds to the highest singular value of the matrix. As Fig. S1b shows, the magnitude of the contribution from the atomistic forces decreases with increasing noise levels. This is intuitive as the contribution from the zero-noise atomistic system should become negligible when all beads are mixed together by a large noise level.

B. Model training

MLCG models were calibrated on coordinates and forces generated via all-atom simulations. Training was performed either using the same procedure as in previous work [7] (Eq. (1)), against denoising scores (Eq. (S4)), or using a linear combination of force and noise information (Eq. 4). When κ was used, coordinates were noised and forces were replaced by the corresponding terms during the creation of each batch and used to define the least-squares regression loss. For an objective comparison of the impact of the different noise levels and force-definition strategies, other factors are held constant; these factors included model hyperparameters (e.g., SchNet architecture, the fitting procedure of prior terms), training setups (e.g., batch size, learning rate) as well as the training-validation splits.

While the Chignolin, TrpCage, and NTL9 models make use of an amino acid-dependent embeddings, the Ubiquitin model makes use of a sequence-positional embedding. Following [22], the Ubiquitin models were also trained using decoy frames at noise levels σ of 0.5 Å and 5.0 Å for 2% of the training data to stabilize extrapolation to high energy configurations.

TABLE S1. Epochal checkpoints chosen for Trp-Cage. The value for force & noise and noise-only models are on the left and right of the slash for non-vanishing noise levels, respectively.

Selected Epochs	Percentage of training set used / Epochal saving stride				
Noise level (Å^2)	100%/1	20%/1	5%/2	2%/5	1%/10
0	158[b]	17[b]	33[b]	19[b]	19[b]
0.0005	199[a]/84[b]	49[b]/31[b]	27[b]/39[b]	29[b]/19[b]	29[b]/39[b]
0.003	199[a]/146[b]	199[a]/61[b]	89[b]/53[b]	59[b]/44[b]	39[b]/49[b]
0.005	199[a]/121[b]	144[b]/181[b]	155[b]/49[b]	164[b]/49[b]	119[b]/59[b]

[a] Picked at fixed epoch after validation loss plateaued.
[b] Picked epoch with lowest validation loss.

1. *Model selection*

A criterion is needed to decide which training epoch should represent the best model associated with each training setup. Since different learning conditions result in different training dynamics and rates of overfitting, selecting the same epoch for models which vary only in the definition of forces does not result in a fair comparison. Nevertheless, we aim to use a unified approach for the model selection such as to reduce subjective bias and random variation. We base epoch selection primarily on picking the model with lowest validation loss. This is possible when validation loss curves exhibit an uptick; this occurs for pure force matching or low noise level training on low-data schemes. However, for cases with abundant training data or a high noise level, no uptick of the validation loss is observed. As a result, in these cases training was stopped at a certain number of epochs (e.g., 200 for NTL9 models on full training data) and the corresponding model was then used for simulation. Note that even after the validation loss plateaued, there were cases where the model quality seemed to fluctuate, or even deteriorate, during continued training despite decreasing validation losses. This was especially observed in NTL9 models at intermediate noise levels (e.g., 0.003 Å^2). This issue was mitigated by selecting an earlier epoch: checkpoints for models trained on 100% and 20% training data were chosen such that the number of optimizer updates was equal to that utilized for the epoch selected for training on 5% data.

In Table S1 and S2 we summarize the epoch numbers selected for the analyses in this work and which strategy was used. We note that it has been observed in previous work that the validation force/score matching loss alone does not guarantee the best model for simulation [23, 24]. The stability of the CG model training and effective assessment of the quality of a CG force-field without extensive simulations are open challenges and future work is needed to study them systematically.

For Ubiquitin, a different approach was used for selecting an optimal model. First, experiments were performed in order to find the optimal SMD/folded ratio of the dataset composition. After determining that 1:1 ratio was the optimal, we subsample the dataset and trained models for 460k (the maximum size given the number of SMD frames), 230k and 153k frames. For each of these dataset sizes, we trained five models: one using only atomistic forces and four others using atomistic and denoising forces with levels of 0.001, 0.003, 0.005 and 0.01 Å^2. All models were trained to epoch 200 and simulated at different epochs with different starting configurations. Figure S2 shows the results from simulations at epoch 80, at which the validation loss stabilizes. Langevin simulations at later epochs showed similar performance.

TABLE S2. Epochal checkpoints chosen for NTL9. The value for force & noise and noise-only models are on the left and right of the slash for non-vanishing noise levels, respectively.

Selected Epochs	Percentage of training set used / Epochal saving stride				
Noise level (Å^2)	100%/1	20%/1	5%/5	2%/5	1%/10
0	199[a]	899[a]	119[b]	44[b]	29[b]
0.0005	179[b]/170[b]	971[b]/449[b]	359[b]/239[b]	69[b]/74[b]	59[b]/49[b]
0.003	54[c]/196[b]	275[c]/564[b]	1104[b]/1434[b]	854[b]/854[b]	229[b]/199[b]
0.005	199[a]/196[b]	899[a]/823[b]	1454[b]/1434[b]	2429[b]/854[b]	299[b]/269[b]

[a] Picked at fixed epoch after validation loss plateaued.
[b] Picked epoch with lowest validation loss.
[c] Picked with comparable training batch count as the epoch picked at stride 20.

2. Convergence of model simulations

For model quality assessment, we compare the FES of the CG model with the FES of corresponding all-atom simulations. The FES of the CG model is estimated by the histogram of long simulations. These simulations were initialized from configurations randomly selected from the atomistic data sets, providing a diverse set of starting points across each FES.

Trp-Cage Each Trp-Cage model was simulated for 5M time steps (2 fs each) from 100 starting structures randomly sampled from the training dataset. The first 40% of each independent trajectory (2M steps) was discarded, while the rest was aggregated and converted to a histogram; $-\log$ of the histogram frequencies corresponds to the FES as visualized in the main text.

In order to demonstrate the convergence of the simulations, we split each trajectory into 10 non-overlapping chunks in time, each spanning 0.5M time steps. In Fig. S3a an example is provided for noise level 0.003 Å^2 trained on 2% of available training data. We observed that after the initial shift of the distribution, the later chunks all lie within a small level of variation without systematic drift. There are frequent transitions between the folded and unfolded states in each trajectory (Figure S3b). The C_α-RMSD curve and the TIC curve outline the same trends, with short residence time in positive TIC 1 and small RMSD corresponding to the folded state. This coincides with the observation in Fig. S3a and main text that the model is underestimating the stability of the folded state for this training set size and optimization procedure.

NTL9 For NTL9 similar analyses were conducted in Fig. S4. Owing to the slower kinetics, we observed a gradual shift of the distribution on TIC 1 over time, which reflects the evolution of the 100-replica ensemble from the biased starting distribution. After a longer burn-in period (curves in blue, cyan and yellow), evolution along the FES converges, indicating that the simulations together are sampling the Boltzmann distribution. Figure S4b shows the TIC time series of 5 randomly picked trajectories, demonstrating bidirectional transitions between the folded and unfolded states. The RMSD curves are omitted for visual clarity.

Ubiquitin For Ubiquitin, the convergence of the PT simulations was assessed by checking the invariance of the RMSD distribution as a function of time. Beyond the transition region, which is lost at the beginning of the simulation, the distributions for different time windows over the whole simulation time are similar, showing a stable folded and unfolded basin. Furthermore, tracing individual replicas across temperatures shows that bidirectional crossings between the folded and unfolded basins during the course of the simulation (Figure S5 b.). For Figure 4, the first 6M frames of the simulation were discarded as burn-in.

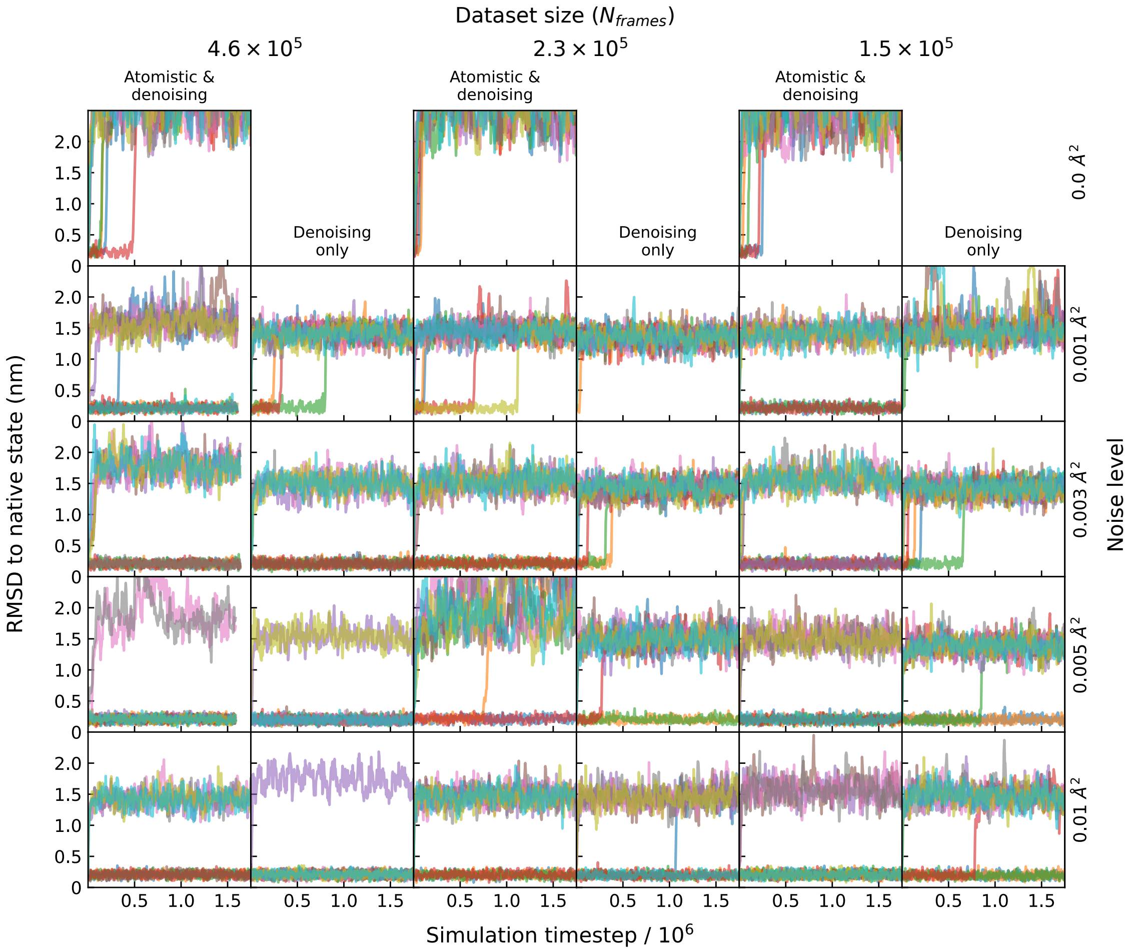

FIG. S2. UBQ model selection. 1-D RMSD traces of Langevin simulations at 300K from models trained with different dataset size and different noise levels. Top row corresponds to force-only models which are unable to stabilize the folded state.

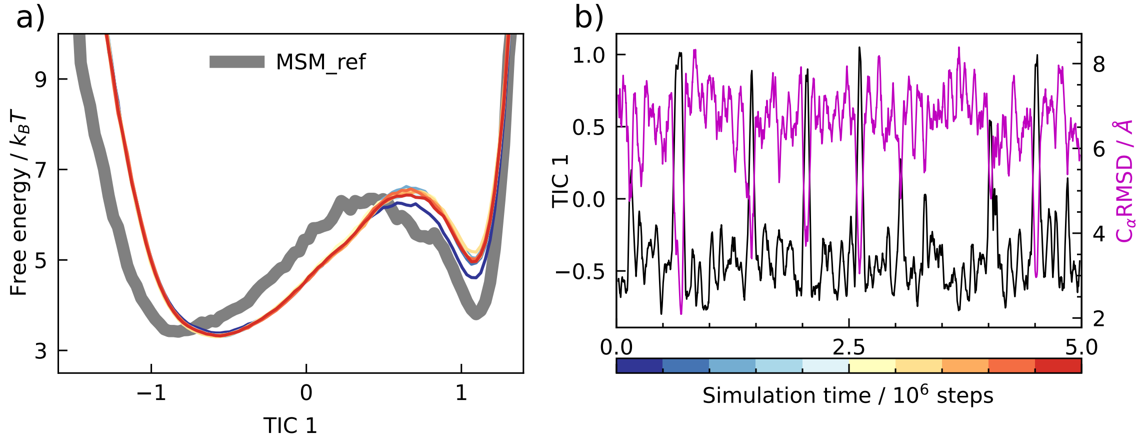

FIG. S3. Convergence of simulations for a Trp-Cage model. a. 1-D histogram over the first TIC for models trained with a combination of denoising forces and atomistic forces on 2% training set at noise level 0.003 Å^2. Aggregated distribution from all 100 replicas in each of the 10 time windows is visualized with color reflecting the time frame. The reference FES is shown in gray. b. 1-D time series of a single trajectory of the above simulation projected on the first TIC and RMSD.

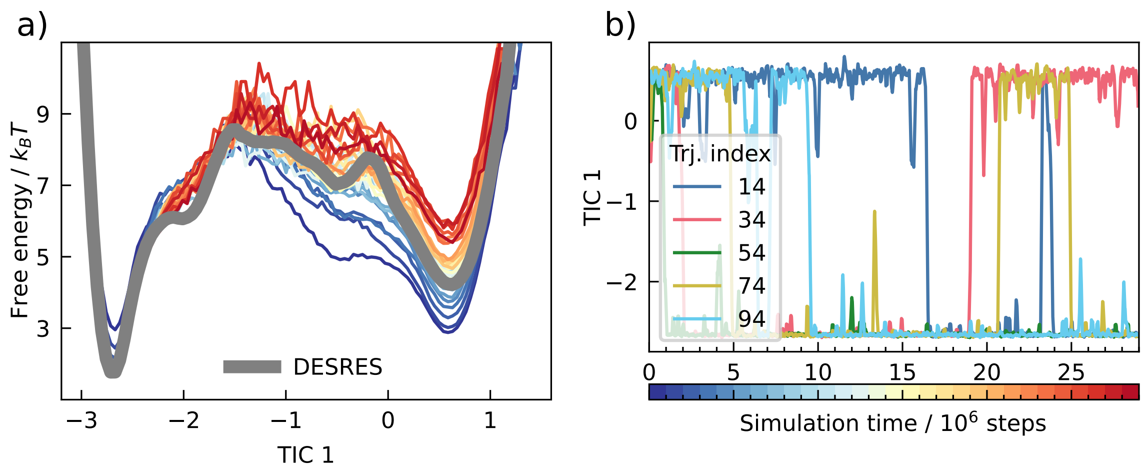

FIG. S4. Convergence of simulations for an NTL9 model. a. 1-D histogram over the first TIC for models trained with a combination of denoising forces and atomistic forces on 2% training set and noise level 0.003 Å^2. Aggregated distribution from all 100 replicas in each time window is visualized according to the color bar. The reference FES is shown in gray. b. 1-D time series of five trajectories of the above simulation projected on the first TIC.

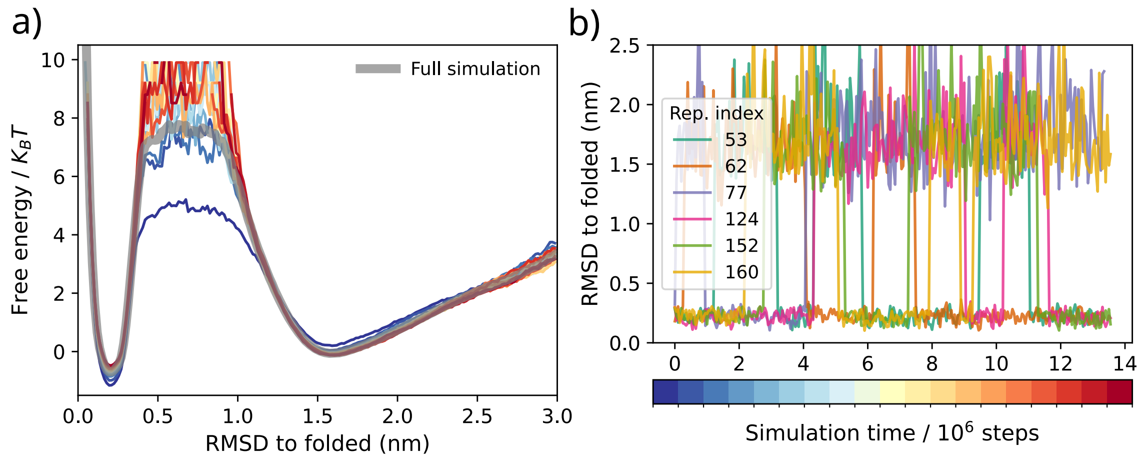

FIG. S5. Convergence of simulations for UBQ models. a. 1-D histogram over the RMSD to the native structure (1D3Z) of PT simulations of a model trained on 230k simulation frames (consisting of 1.25 % of the total folded data and 50 % of the SMD data) using atomistic and denoising forces with a noise level of 0.005 Å^2. b. 1-D RMSD time series of some selected replicas showing mixture of the populations between folded and unfolded across different temperatures.

C. Two-dimensional FESs

Measure of PMF error PMF errors presented in the main text were calculated by creating histograms across the leading two TICs. Bin definitions underlying the histograms were held constant when analyzing simulations of each molecule, and consisted of dividing each TIC axis into 100 equally sized windows between the maximum and minimum values observed across all corresponding models and reference simulations. The proportion of samples present in each window was transformed using $-\log$ and compared to reference values bin-wise using a square loss. This bin-wise loss was averaged using the population present in each bin as weights to create a single number characterizing

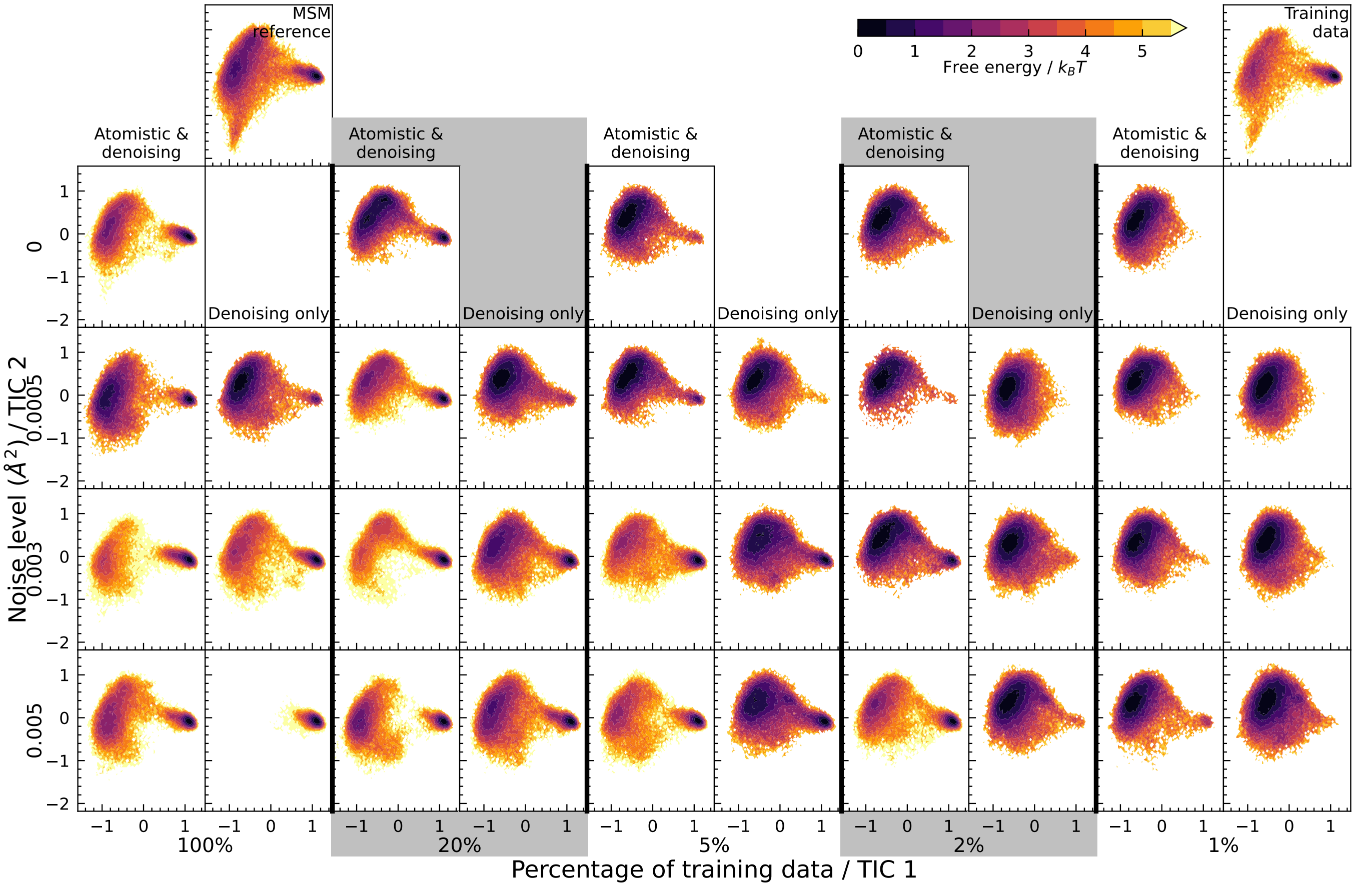

FIG. S6. 2D FESs for Trp-Cage models.

accuracy. Bins without any samples were assigned a baseline proportion of 10^{-6}. Trajectories were trimmed before error calculation using the same procedure as described below for the creation of 2D FES visualizations. The same data are also projected onto C_α–RMSD and R_g, a commonly used combination of observables for comparison.

Trp-Cage The FESs over the first and second TICs for all utilized models are visualized in Fig. S6. As stated in the main text, models trained only on forces in the low-data regime cannot fold Trp-Cage, and the inclusion of noising allows the model to correctly stabilize the folded state. The second TIC resolves mainly the conformational kinetics in the unfolded state, which was preserved among all models that recovered the unfolded state (Fig. S6).

The FESs over RMSD–R_g are similarly visualized in Fig. S7.

NTL9 Corresponding 2D FESs are found for NTL9 in Figure S8. Similar to the Trp-Cage, when focusing on the folded (bottom left) and unfolded (center right) states, the same trend as discussed in the main text is observed. Unlike in the case of Trp-Cage, the second TIC helps to distinguish several misfolded states and folding intermediates. Moreover, we observed that the distribution of those minor states does not converge to the reference ANTON simulations monotonically. We attribute this phenomenon to the fact that the transitions connecting those states are not thoroughly sampled in the atomistic reference despite the extensive simulations, as well as their slightly altered temperature.

The FESs over RMSD–R_g are similarly visualized in Fig. S9.

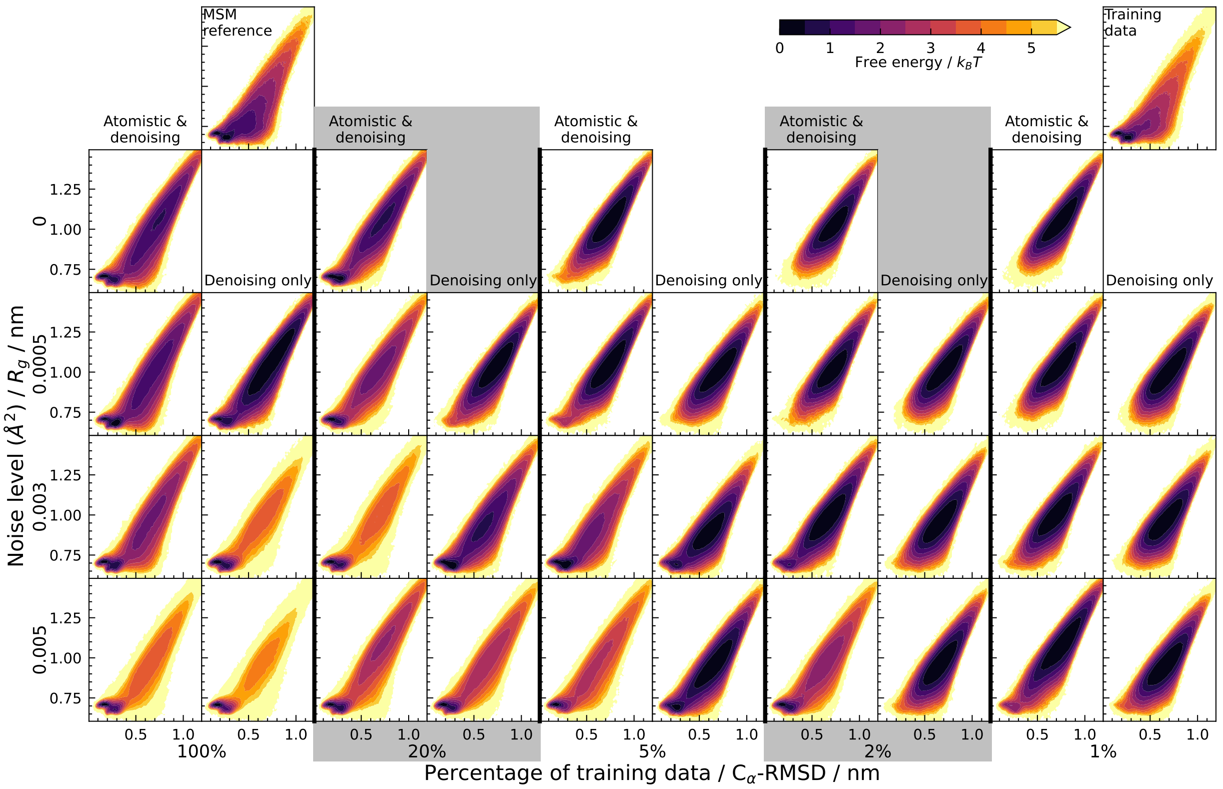

FIG. S7. 2D FESs over RMSD–R_g for Trp-Cage models.

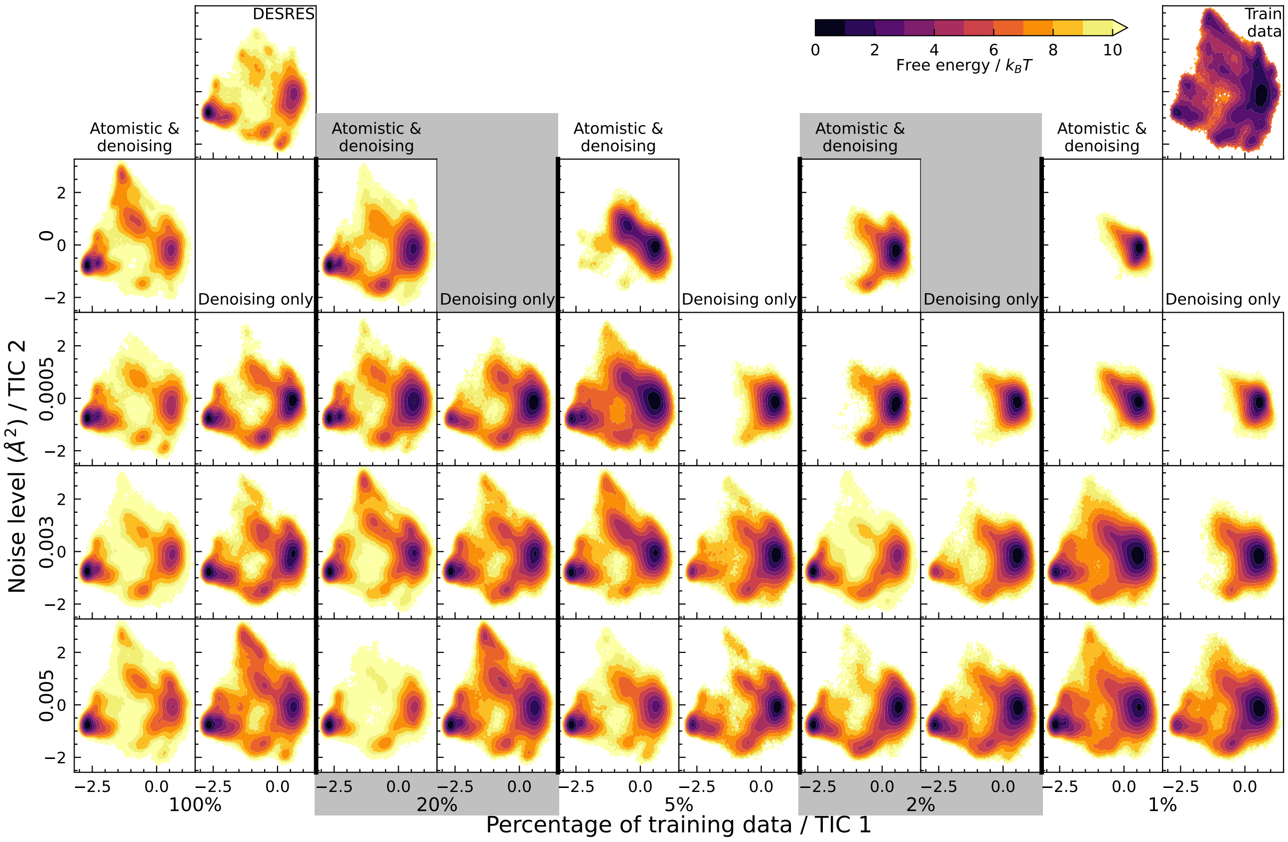

FIG. S8. 2D FESs for NTL9 models.

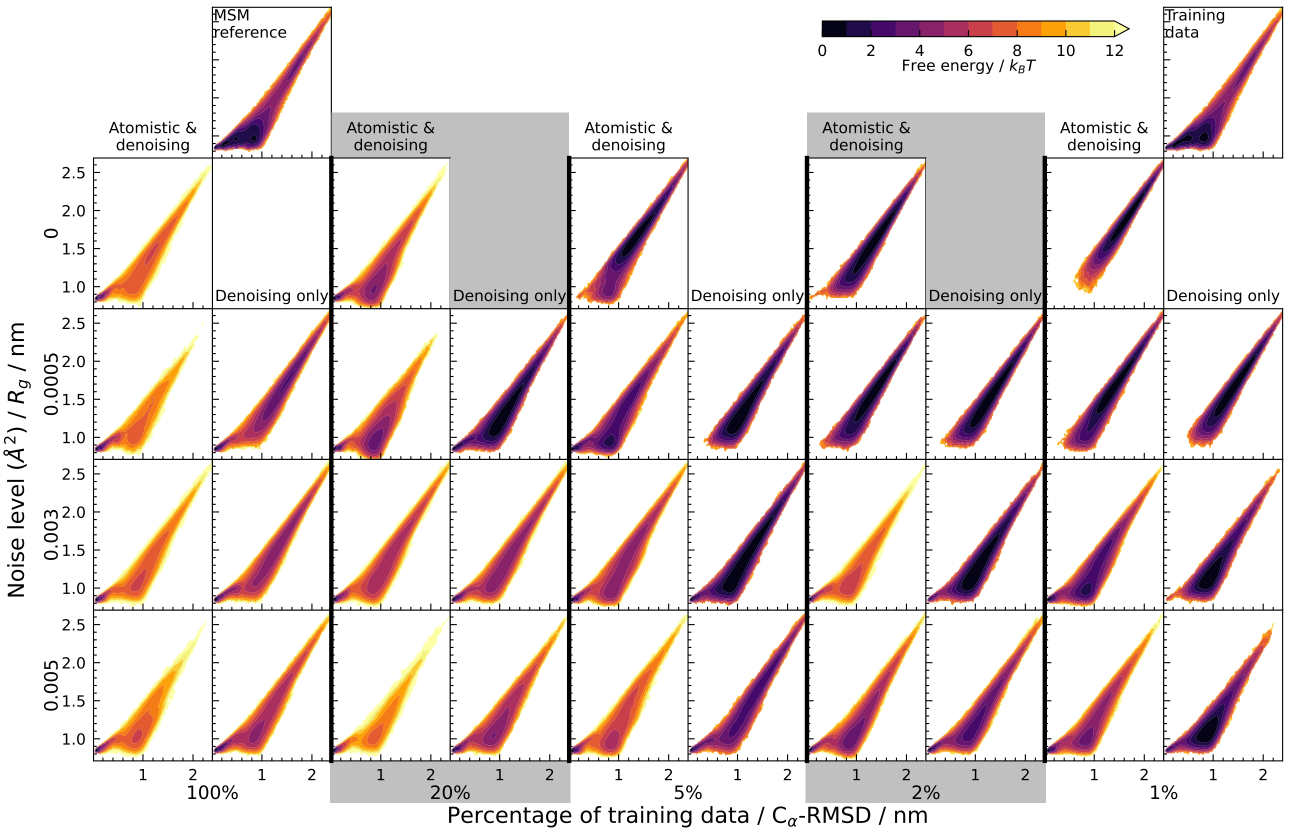

FIG. S9. 2D FESs over RMSD–R_g for NTL9 models.

D. Results for Chignolin

We include here results on a 10-residue miniprotein Chignolin to facilitate comparison with previous work (e.g., [25]). In Ref. [25], the Chignolin model was trained over data provided from DESRES, which consists of 534,743 conformations spanning 106 μs all-atom MD simulations in explicit solvent at 340 K. Since that dataset does not contain atomistic forces, we run experiments on a comparable dataset from Ref. [26], which was extracted from simulations similar to Trp-Cage and NTL9. The dataset consists of around 1.8×10^6 conformations sampled from 3,744 short trajectories at 350 K, adding up to 186 μs aggregated simulation time.

Chignolin was analyzed using a similar approach to that used for TrpCage and NTL9. Fig. S10 shows the free-energy curves along the first TIC that capture the folding-unfolding transition. We find that the proposed method can create models that stabilize the folded state with as little as 1% of the training data, which roughly corresponds to 4% of the dataset used by Ref. [25]. Figs. S11 and S12 illustrate the 2-D free energy surfaces over TICs and RMSD-R_g, respectively. Unlike the examples provided in the main text, the addition of atomistic force information into denoising-based training provides minimal gain; the cause for this is unclear. However, we note that the systems described in the main text better represent possible future applications on complex biomolecules because of their increased size and complexity.

E. Rare-event sampling

For systems with well-converged training data (i.e., Chigolin, Trpcage and NTL9), we demonstrate the capabilities of our models in sampling the transition states as well as reproducing the correct

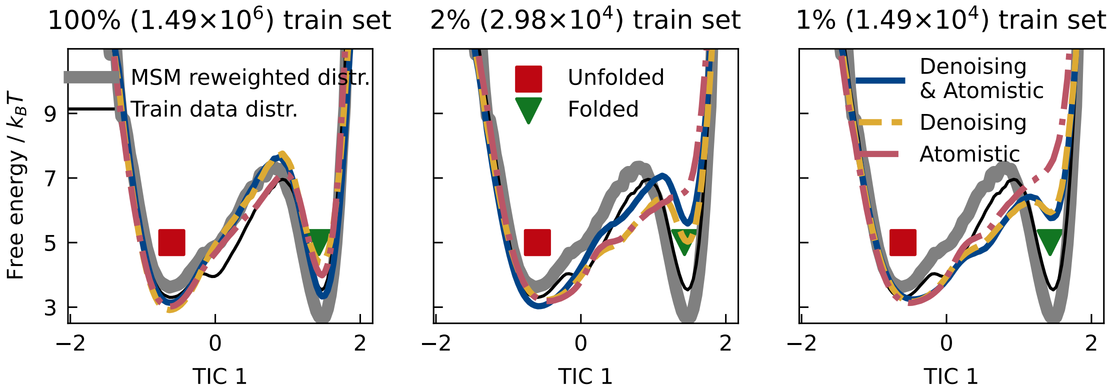

FIG. S10. MLCG model of Chignolin. a. 1-D FES over the first TIC for models trained (I) with a combination of denoising forces and atomistic forces (dark blue), (II) with denoising forces (yellow) or (III) with atomistic forces (red) on different strides of the training dataset. The reference FES is shown in solid gray and training data distribution in thin black. Only noise level 0.003 $Å^2$ is shown.

folding-unfolding pathways. Figure S13 visualizes the fraction of native contacts formed within each local motif as function of the overall fraction of native contacts of the system. We compare the curves for the atomistic reference and three of our models trained with only 1% of the dataset. The models are generally capable of reproducing well the correct folding process for the relevant contact groups with respect to the atomistic reference. We view this as evidence that folding transition states are sampled with the correct combination of partially formed structural motifs. We note that the curves of the force-only model of NTL9 do not reach the region where $Q > 0.7$, as the simulations with this model did not sample the folded state.

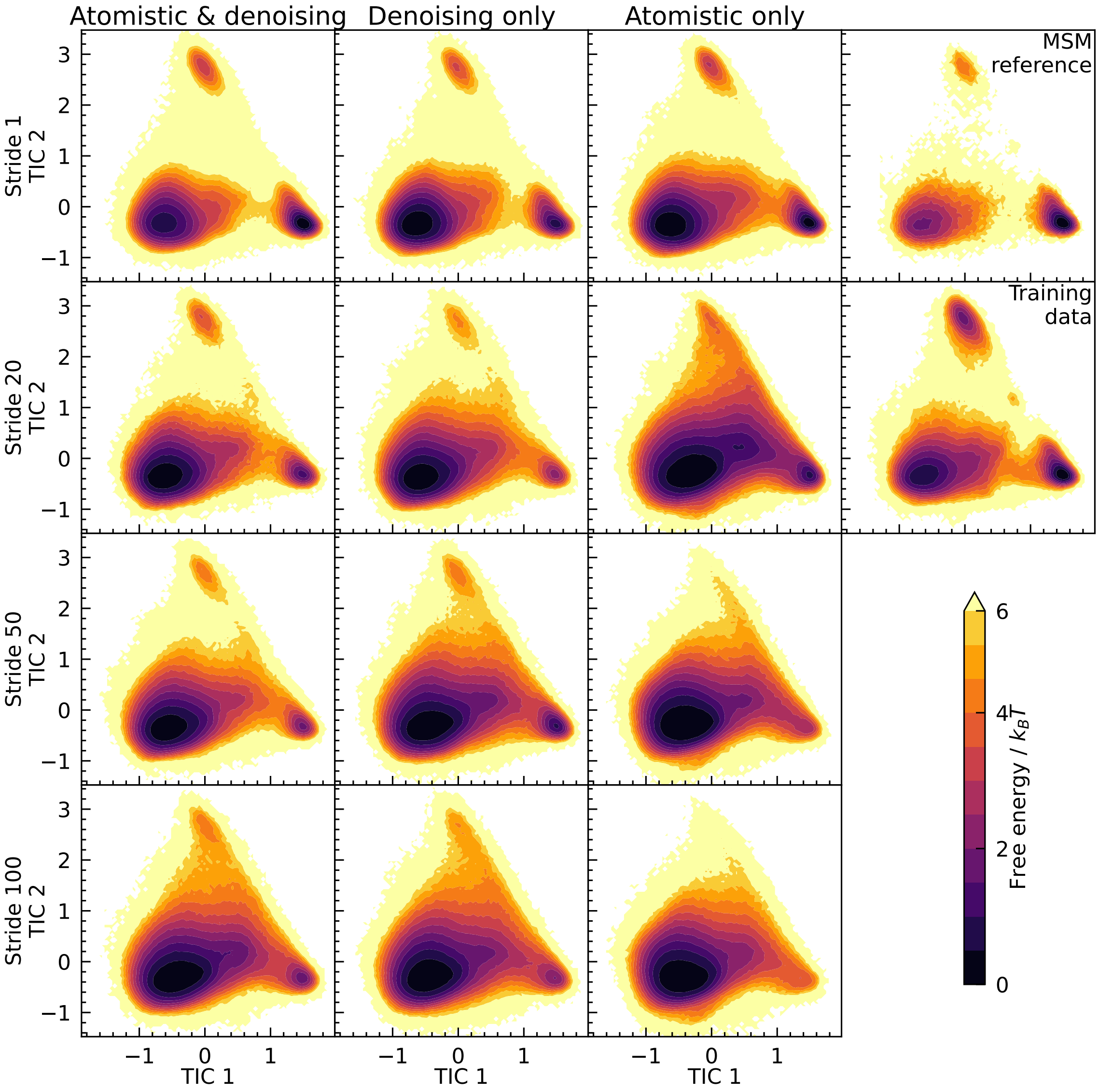

FIG. S11. 2D FESs for Chignolin models.

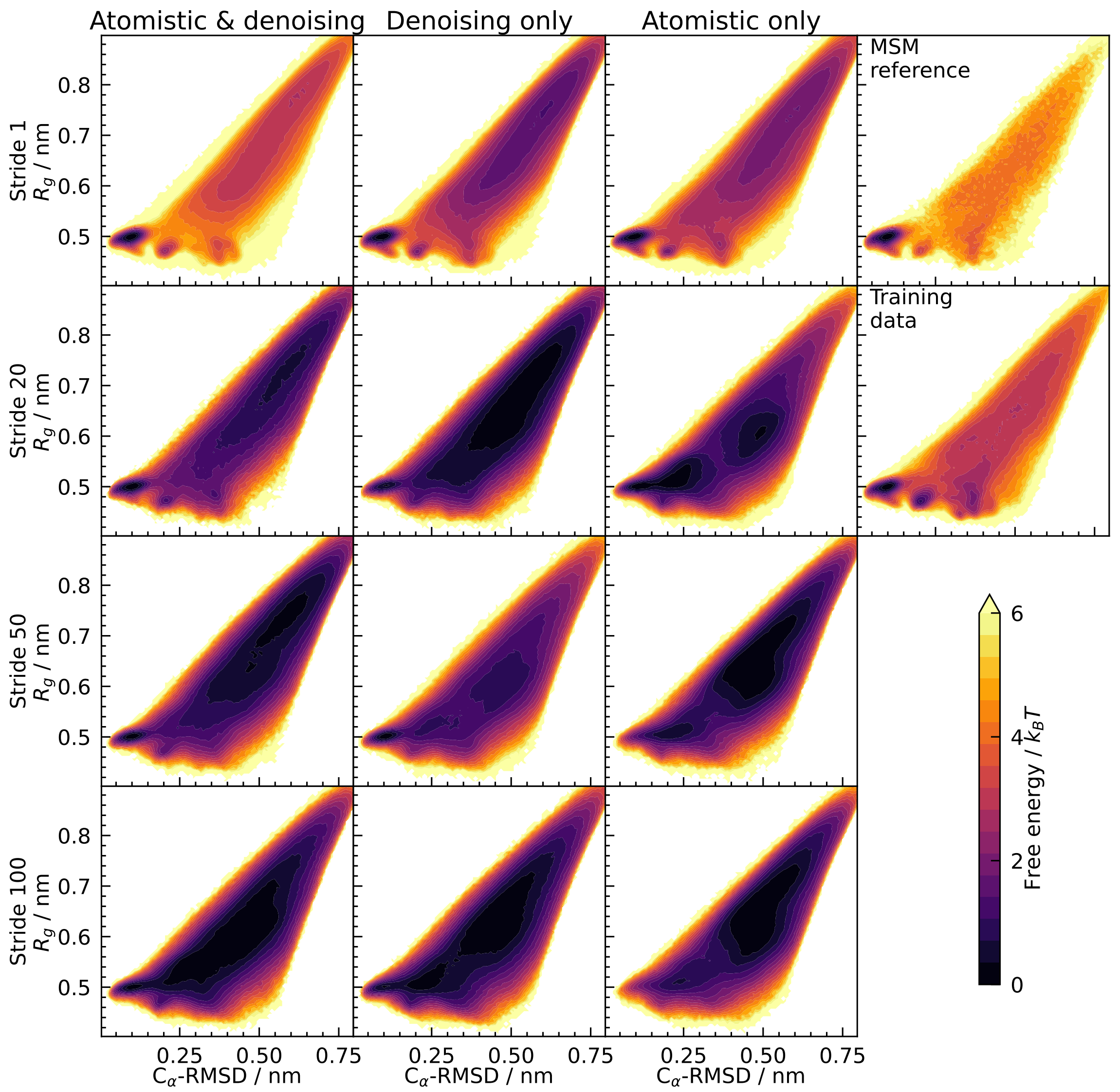

FIG. S12. 2D FESs for Chignolin models over RMSD-R_g.

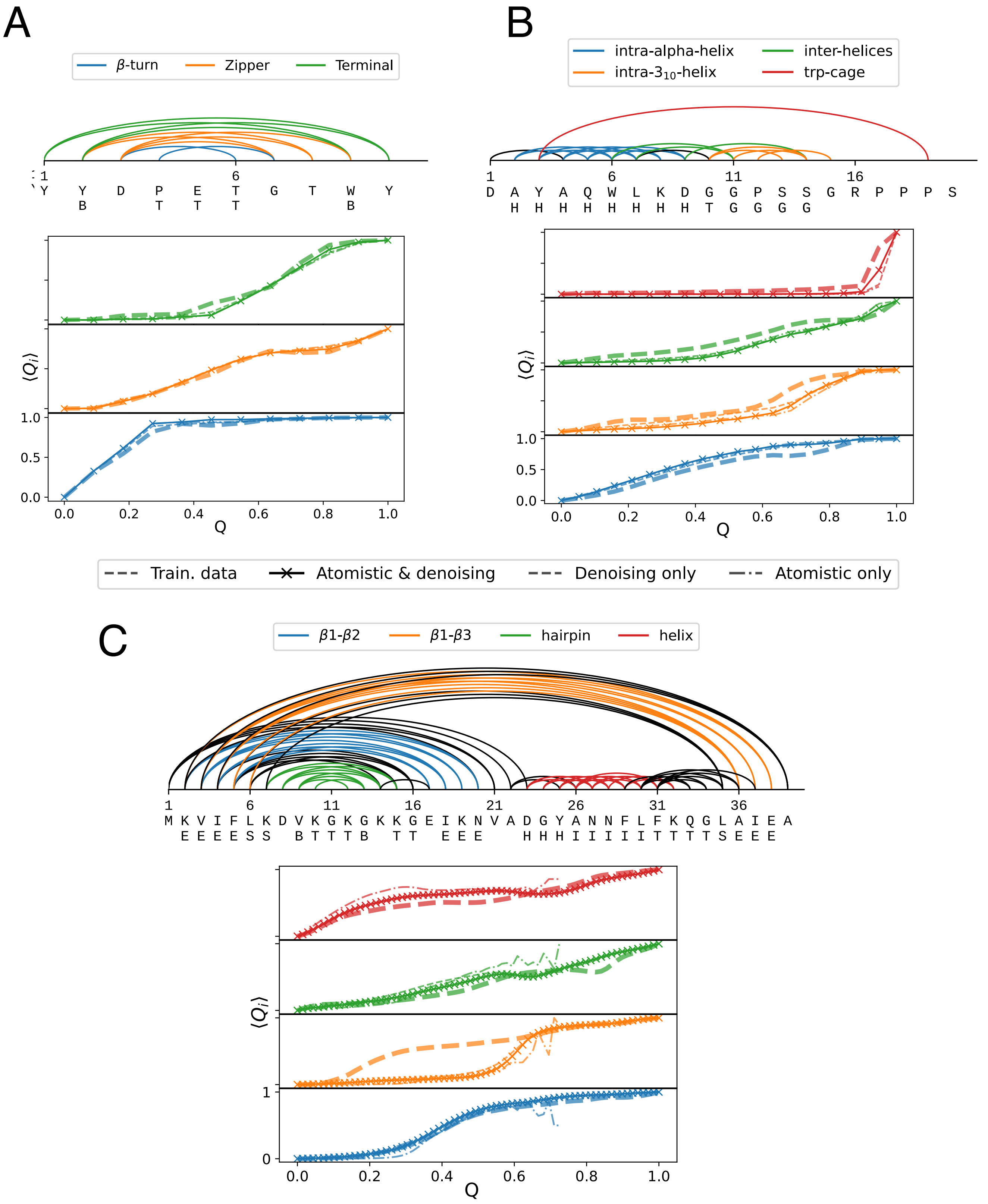

FIG. S13. Fraction of native contacts analyses. (A) Chignolin (B) TRPCage and (C) NTL9 models were trained with 1% of the total training data (c.f. figures 2, 3 and S12). Different colors represent the contacts associated to different structural motifs, which are described in the top of the panels. Bottom of each panel shows average fractions of native contacts $\langle Q_i \rangle$ of each motif grows as function of the total number of native contacts Q.

[1] W. G. Noid, J. W. Chu, G. S. Ayton, V. Krishna, S. Izvekov, G. A. Voth, A. Das, and H. C. Andersen, The multiscale coarse-graining method. i. a rigorous bridge between atomistic and coarse-grained models, J. Chem. Phys. **128**, 244114 (2008).

[2] J. Jin, A. J. Pak, A. E. Durumeric, T. D. Loose, and G. A. Voth, Bottom-up coarse-graining: Principles and perspectives, J. Chem. Theory Comput. **18**, 5759 (2022).

[3] W. G. Noid, Perspective: Advances, challenges, and insight for predictive coarse-grained models, J. Phys. Chem. B **127**, 4174 (2023).

[4] G. Ciccotti, T. Lelievre, and E. Vanden-Eijnden, Projection of diffusions on submanifolds: Application to mean force computation, Commun. Pure Appl. Math. **61**, 371 (2008).

[5] E. Kalligiannaki, V. Harmandaris, M. A. Katsoulakis, and P. Plecháč, The geometry of generalized force matching and related information metrics in coarse-graining of molecular systems, J. Chem. Phys. **143** (2015).

[6] G. Ciccotti, R. Kapral, and E. Vanden-Eijnden, Blue moon sampling, vectorial reaction coordinates, and unbiased constrained dynamics, ChemPhysChem **6**, 1809 (2005).

[7] A. Krämer, A. E. Durumeric, N. E. Charron, Y. Chen, C. Clementi, and F. Noé, Statistically optimal force aggregation for coarse-graining molecular dynamics, J. Chem. Phys. Lett. **14**, 3970 (2023).

[8] G. M. Torrie and J. P. Valleau, Nonphysical sampling distributions in monte carlo free-energy estimation: Umbrella sampling, J. Comput. Phys. **23**, 187 (1977).

[9] L. Rosso, P. Mináry, Z. Zhu, and M. E. Tuckerman, On the use of the adiabatic molecular dynamics technique in the calculation of free energy profiles, J. Chem. Phys. **116**, 4389 (2002).

[10] L. Maragliano and E. Vanden-Eijnden, A temperature accelerated method for sampling free energy and determining reaction pathways in rare events simulations, Chem. Phys. Lett. **426**, 168 (2006).

[11] T. Lelièvre, M. Rousset, and G. Stoltz, Computation of free energy profiles with parallel adaptive dynamics, J. Chem. Phys. **126** (2007).

[12] A. Lesage, T. Lelièvre, G. Stoltz, and J. Hénin, Smoothed biasing forces yield unbiased free energies with the extended-system adaptive biasing force method, J. Phys. Chem. B **121**, 3676 (2017).

[13] T. Hastie, R. Tibshirani, J. H. Friedman, and J. H. Friedman, *The elements of statistical learning: data mining, inference, and prediction*, Vol. 2 (Springer, 2009).

[14] C. M. Bishop and N. M. Nasrabadi, *Pattern recognition and machine learning*, Vol. 4 (Springer, 2006).

[15] W. Noid, J.-W. Chu, G. S. Ayton, and G. A. Voth, Multiscale coarse-graining and structural correlations: Connections to liquid-state theory, J. Phys. Chem. B **111**, 4116 (2007).

[16] M. S. Shell, The relative entropy is fundamental to multiscale and inverse thermodynamic problems, J. Chem. Phys. **129**, 144108 (2008).

[17] J. Köhler, Y. Chen, A. Krämer, C. Clementi, and F. Noé, Flow-matching: Efficient coarse-graining of molecular dynamics without forces, J. Chem. Theory Comput. **19**, 942 (2023).

[18] B. E. Husic, N. E. Charron, D. Lemm, J. Wang, A. Pérez, M. Majewski, A. Krämer, Y. Chen, S. Olsson, G. D. Fabritiis, F. Noé, and C. Clementi, Coarse graining molecular dynamics with graph neural networks, J. Chem. Phys. **153**, 194101 (2020).

[19] J. Mullinax and W. Noid, Generalized yvon-born-green theory for molecular systems, Phys. Rev. Lett. **103**, 198104 (2009).

[20] J. F. Rudzinski and W. Noid, Coarse-graining entropy, forces, and structures, J. Chem. Phys. **135** (2011).

[21] A. Durumeric, Y. Chen, A. S. Pasos-Trejo, A. Kraemer, and E. Rolando, noegroup/aggforce: 1.0.1 (2026).

[22] N. E. Charron, K. Bonneau, A. S. Pasos-Trejo, A. Guljas, Y. Chen, F. Musil, J. Venturin, D. Gusew, I. Zaporozhets, A. Krämer, C. Templeton, A. Kelkar, A. E. P. Durumeric, S. Olsson, A. Pérez, M. Majewski, B. E. Husic, A. Patel, G. De Fabritiis, F. Noé, and C. Clementi, Navigating protein landscapes with a machine-learned transferable coarse-grained model, Nat. Chem. **17**, 1284–1292 (2025).

[23] X. Fu, Z. Wu, W. Wang, T. Xie, S. Keten, R. Gomez-Bombarelli, and T. Jaakkola, Forces are not enough: Benchmark and critical evaluation for machine learning force fields with molecular simulations, arXiv preprint arXiv:2210.07237 (2022).

[24] B. R. Duschatko, J. Vandermause, N. Molinari, and B. Kozinsky, Uncertainty driven active learning of coarse grained free energy models, Npj Comput. Mater. **10**, 9 (2024).

[25] J. Zhang, X. Lin, Y. Q. Gao, *et al.*, Invertible coarse graining with physics-informed generative artificial intelligence, arXiv preprint arXiv:2305.01243 (2023).

[26] M. Majewski, A. Pérez, P. Thölke, S. Doerr, N. E. Charron, T. Giorgino, B. E. Husic, C. Clementi, F. Noé, and G. De Fabritiis, Machine learning coarse-grained potentials of protein thermodynamics, Nat. Commun. **14** (2023).
